# Breaking the Temperature Barrier: Lasting Pain Relief and Motor Recovery After 80°C Suprascapular Nerve Ablation

**DOI:** 10.7759/cureus.89729

**Published:** 2025-08-10

**Authors:** Jad Kabbara, Cham Alsalak, Abdallah Kabbara

**Affiliations:** 1 Anesthesiology, Lake Erie College of Osteopathic Medicine, Westlake, USA; 2 General Medicine, Damascus University, Damascus, SYR; 3 Anesthesiology, University Hospitals Cleveland Medical Center, Westlake, USA

**Keywords:** fluoroscopic guided, radio-frequency ablation, suprascapular nerve block, suprascapular nerve blocks, suprascapular nerve pulsed radiofrequency

## Abstract

Chronic shoulder pain can severely impact function and quality of life. The suprascapular nerve, which innervates most of the shoulder joint, is a key target in refractory cases. This report describes the case of an 87-year-old female with bilateral osteoarthritis who failed conservative treatments. A diagnostic suprascapular nerve block provided complete relief. She then underwent fluoroscopically guided conventional radiofrequency ablation (RFA) at 80°C for 90 seconds, resulting in immediate and sustained pain relief and improved mobility. The pain relief lasted for over two years. This report demonstrates the long-term effectiveness and safety of conventional RFA in managing chronic suprascapular nerve-related shoulder pain.

## Introduction

Chronic shoulder pain is a very common musculoskeletal complaint, with up to 26% of the general public reporting shoulder pain at any given time [1]. This can lead to reduced quality of life, difficulty completing tasks, and overall decreased productivity. Among the various contributors to shoulder pain, the most prevalent is the suprascapular nerve. This nerve innervates roughly 70% of the shoulder joint, including the supraspinatus and infraspinatus muscles, which help with shoulder abduction and external rotation [2]. The suprascapular nerve has been linked to up to 2% of all shoulder pain cases, with a heavy prevalence in athletic populations, accounting for 33% of shoulder pain causes [3].

Many conservative treatments have been attempted to manage chronic shoulder pain, including nonsteroidal anti-inflammatory drugs (NSAIDs), corticosteroid injections, and physical therapy. Unfortunately, these modalities tend to provide only temporary and limited pain relief. This has led to the emergent use of radiofrequency ablation (RFA) for longer-term pain management. A study conducted in 2024 showed that RFA had a clear edge in treating long-term pain compared with suprascapular nerve blocks [4]. This report presents the case of a patient who experienced extended pain relief of over two years following a right-sided fluoroscopic-guided heat lesion RFA of the suprascapular nerve. It also highlights the improvement in motor function using 80°C ablation, which challenges the traditional use of 42°C for motor preservation.

## Case presentation

**Figure 1:**
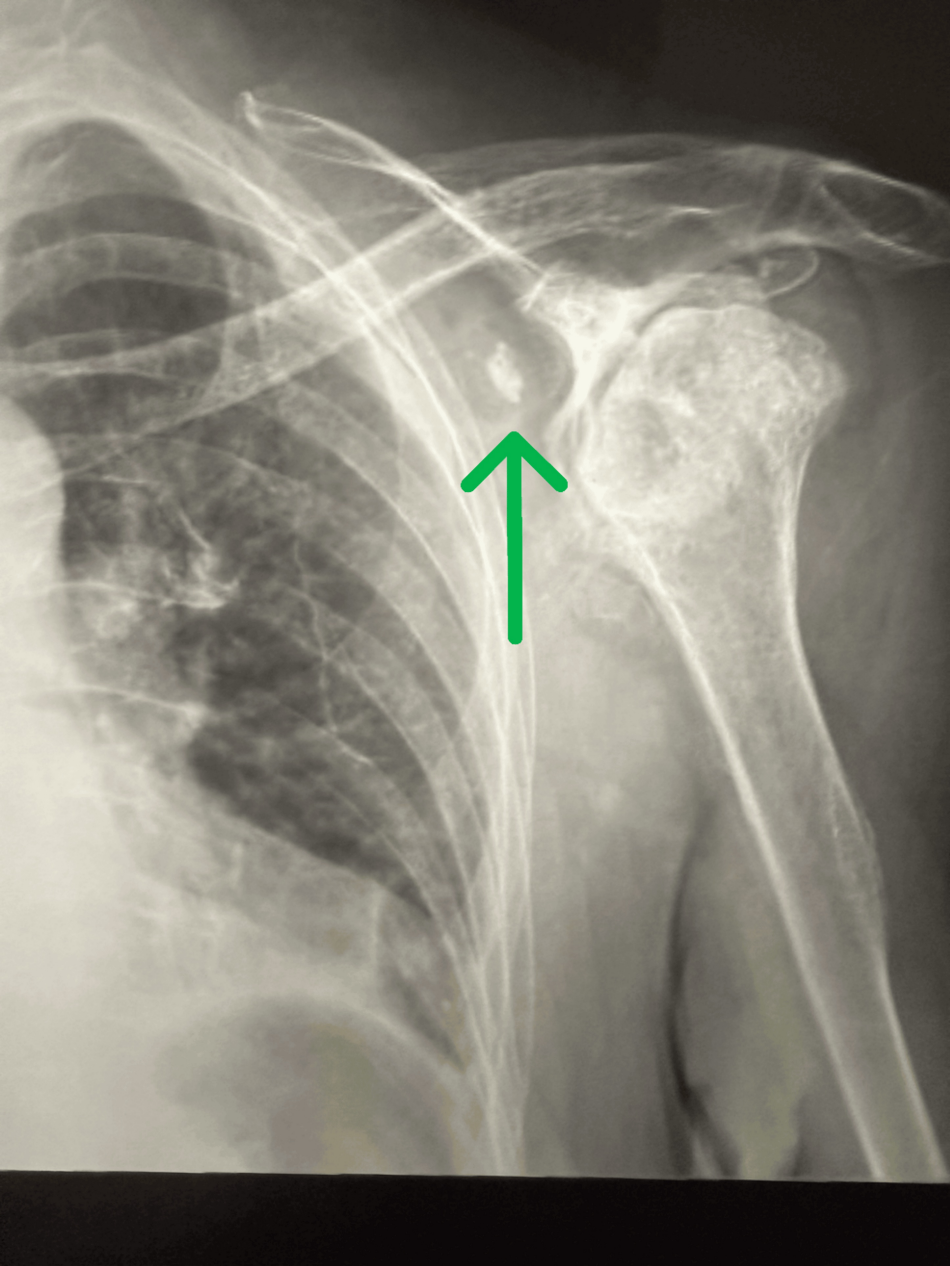
Left shoulder showing severe osteoarthritic changes with multiple loose bodies (green arrow)

**Figure 2:**
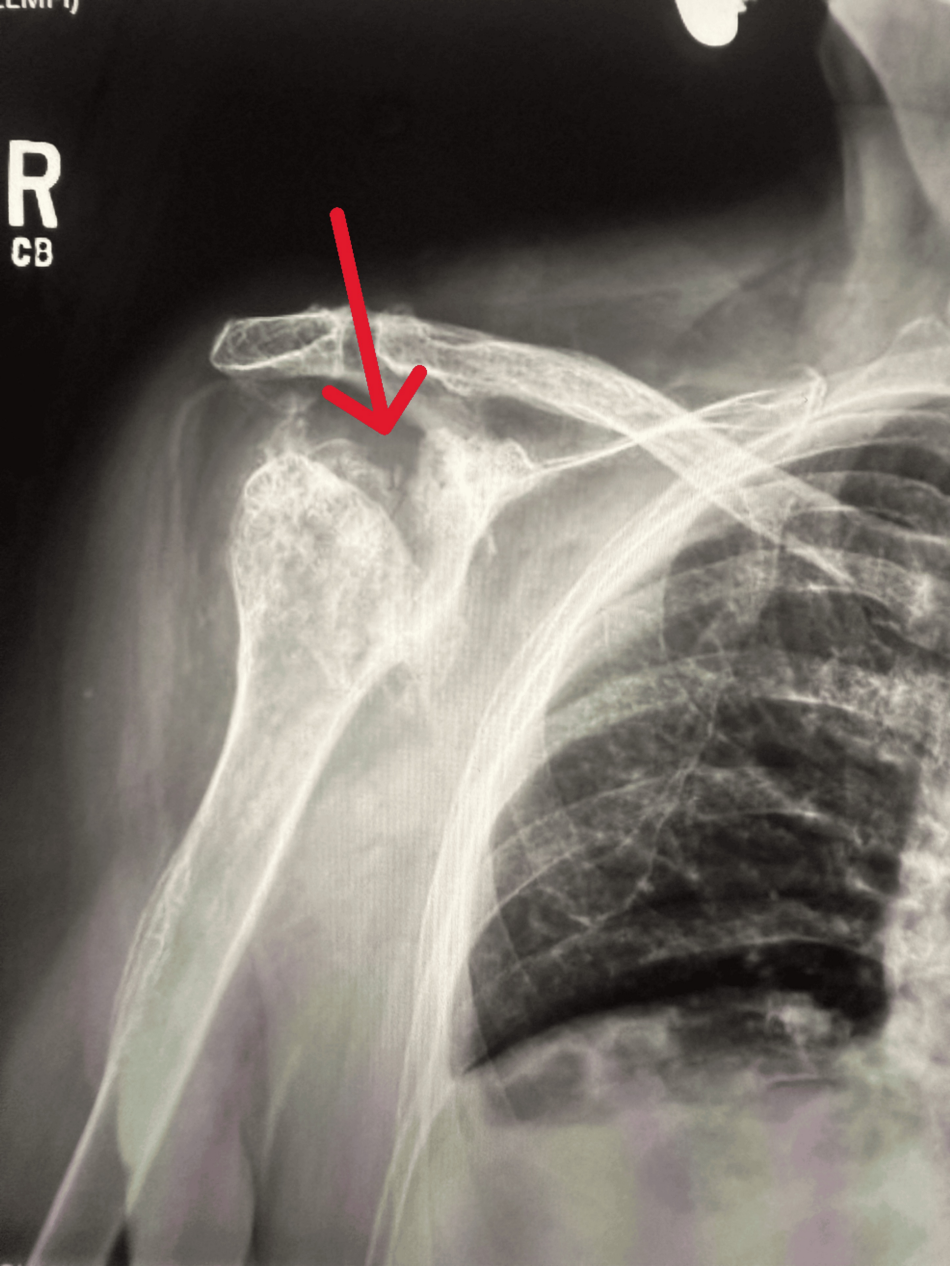
Right shoulder demonstrating progression of glenohumeral joint osteoarthritis, significant flattening of the humeral head, and severe joint space narrowing (red arrow)

An 87-year-old female presented to the pain clinic with severe bilateral shoulder pain and marked restriction of movement, especially on the right side. Bilateral shoulder X-rays revealed advanced degenerative changes in both joints. The left shoulder showed severe osteoarthritic changes with multiple loose bodies (Figure 1), while the right shoulder demonstrated progression of glenohumeral joint osteoarthritis (Figure 2), significant flattening of the humeral head, and severe joint space narrowing. No acute fractures or dislocations were identified.

Conservative treatment, including physical therapy and nonsteroidal anti-inflammatory therapy, had previously failed. The patient was offered a diagnostic suprascapular nerve block under fluoroscopic guidance, which resulted in near-complete improvement in her pain and a significant increase in range of motion. She had initially rated her pain as 10 out of 10. Following the diagnostic nerve block, her pain decreased to 0-1 out of 10, accompanied by significant improvement in joint mobility.

**Figure 3:**
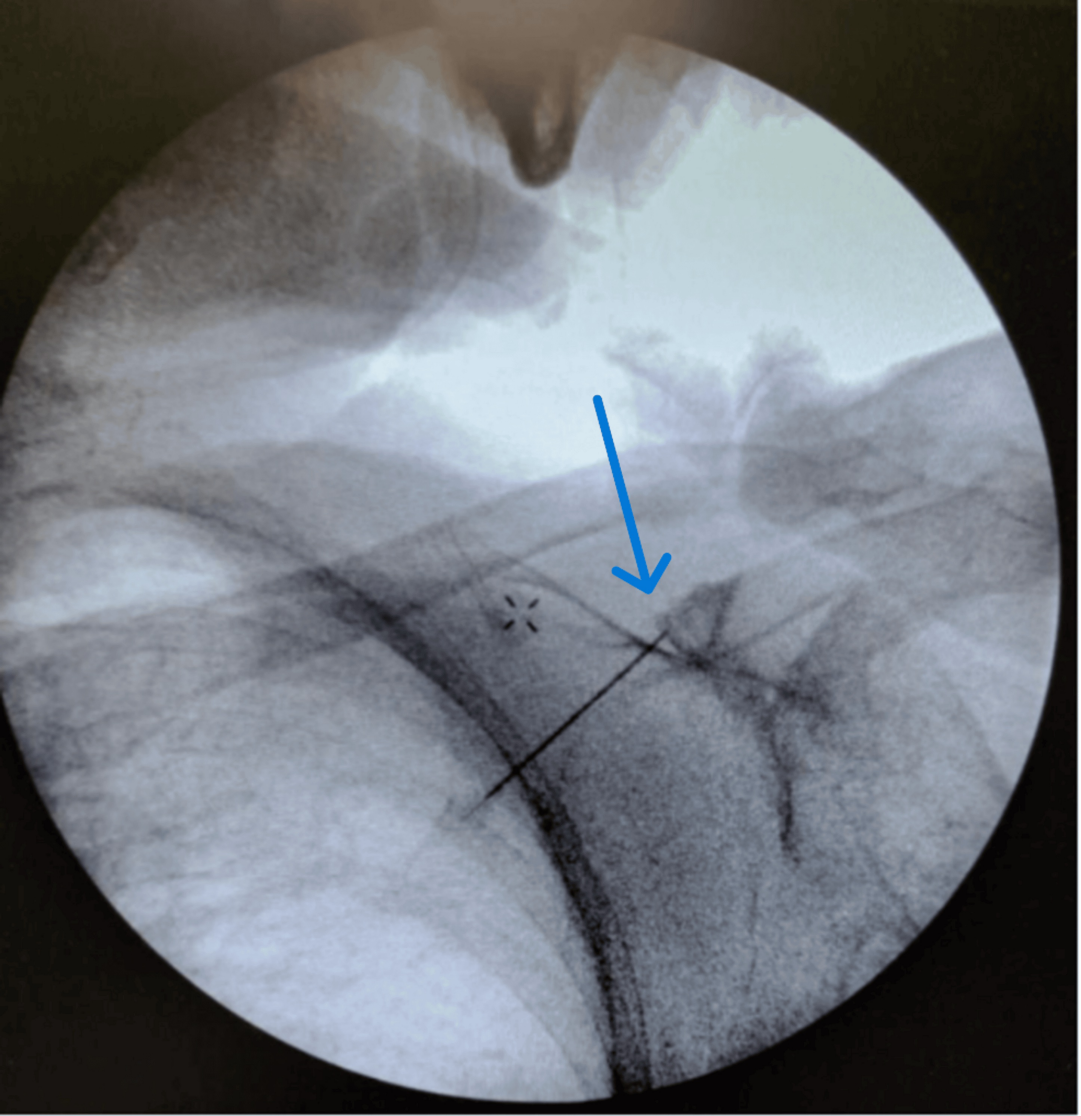
Fluoroscopic image of the right shoulder demonstrating placement of the radiofrequency needle into the suprascapular notch for ablation at 80°C (blue arrow)

Given the positive response, a decision was made to proceed with RFA. RFA of the right suprascapular nerve was performed under fluoroscopic guidance by first identifying the suprascapular notch and anesthetizing the overlying skin and subcutaneous tissue using 1% lidocaine. A 20-gauge radiofrequency needle was advanced into the right suprascapular notch under continuous fluoroscopic visualization (Figure 3). Sensory stimulation at 50 Hz reproduced the patient’s typical symptoms at a threshold below 0.6 V, confirming accurate needle placement. Motor stimulation up to 2 V produced no motor movement. Given the absence of motor response during stimulation, confirming that motor fibers were not in proximity, a decision was made to proceed with thermal lesioning at 80°C for 90 seconds to minimize the risk of motor nerve damage.

The patient tolerated the procedure well and, within days, reported significant improvements in shoulder motion and substantial pain reduction (rated 0-1 out of 10). She was followed up in the pain clinic for two years and continued to demonstrate marked improvements in both shoulder pain and functional mobility.

While previous case reports describe the use of pulsed mode RFA for suprascapular nerve management, this case is unique in that it demonstrates the successful use of conventional thermal RFA at 80°C [5]. Traditionally, high temperatures are avoided due to concern over potential motor nerve damage. However, our patient initially presented with debilitating shoulder pain and complete loss of right shoulder mobility, both of which improved significantly following the RFA. This outcome highlights the potential utility of RFA at 80°C in carefully selected patients and challenges the assumption that only lower temperatures are safe and effective for suprascapular nerve interventions.

## Discussion

Chronic shoulder pain is a significant clinical challenge, particularly in elderly patients with degenerative joint disease or limited surgical options. When conservative therapies fail, the suprascapular nerve becomes a key interventional target due to its substantial role in shoulder innervation and pain transmission. The nerve supplies approximately 70% of the sensory fibers to the shoulder joint and is frequently implicated in both mechanical and inflammatory shoulder conditions [2].

Traditional treatments such as physical therapy, NSAIDs, and corticosteroid injections often yield only short-term benefits. In refractory cases, RFA offers a minimally invasive option with increasing evidence for efficacy in managing chronic shoulder pain [6]. However, most studies in the literature favor pulsed RFA, which modulates pain transmission without fully ablating the nerve, as it is presumed safer for the motor fibers of the supraspinatus and infraspinatus muscles [7]. Our patient’s loss of active shoulder motion eliminated the need to preserve motor function, enabling the safe use of conventional thermal RFA at 80°C for 90 seconds. Before lesioning, motor stimulation confirmed the absence of motor response, ensuring that no functional motor fibers would be affected. The use of fluoroscopic guidance to precisely identify the suprascapular notch further increased the safety and efficacy of the procedure [8].

The most remarkable aspect of this case is the long duration of pain relief - over two years - which significantly exceeds typical outcomes. Literature reports average relief durations of 6-12 months after pulsed RFA. The sustained benefit in this case may be attributed to complete thermal lesioning of the sensory branches, Precise anatomical needle placement, and the chronic nature of the disease, which likely made the nerve more susceptible to lasting disruption. This report underscores the importance of rigorous patient selection, including diagnostic nerve blocks, intraoperative stimulation testing, and precision targeting using imaging guidance. It also supports the growing view that conventional RFA can be used not just as a last resort, but as a primary pain-relief modality in patients who are not candidates for surgery or who seek durable nonsurgical alternatives.

## Conclusions

This report highlights the long-term efficacy of fluoroscopy-guided conventional RFA of the right suprascapular nerve in an elderly patient with refractory shoulder osteoarthritis. By implementing an 80°C thermal ablation, this intervention achieved over two years of sustained pain relief and improved mobility, far exceeding the typical outcome of RFA. The success of this case highlights the value of thorough patient selection, which, when combined with precise anatomical targeting and intraoperative stimulation, can help optimize the results. RFA should be considered as a viable and superior option in managing chronic shoulder pain, especially in those with limited shoulder function.
